# Association of antecedent cardiovascular risk factor levels and trajectories with cardiovascular magnetic resonance-derived cardiac function and structure

**DOI:** 10.1186/s12968-020-00698-w

**Published:** 2021-01-04

**Authors:** Roberto Lorbeer, Susanne Rospleszcz, Christopher L. Schlett, Sophia D. Rado, Barbara Thorand, Christa Meisinger, Wolfgang Rathmann, Margit Heier, Ramachandran S. Vasan, Fabian Bamberg, Annette Peters, Wolfgang Lieb

**Affiliations:** 1 Department of Radiology, University Hospital, LMU Munich, Pettenkoferstr. 8a, 80336 Munich, Germany; 2 German Center for Cardiovascular Disease Research (DZHK E.V.), Munich, Germany; 3 Institute of Epidemiology, Helmholtz Zentrum München, Neuherberg, Germany; 4 Chair of Epidemiology, Institute of Medical Information Processing, Biometrics and Epidemiology (IBE), Faculty of Medicine, LMU Munich, Munich, Germany; 5 Department of Diagnostic and Interventional Radiology, Medical Center, University Freiburg, Freiburg, Germany; 6 Department of Diagnostic and Interventional Radiology, Eberhard Karl University Tübingen, Tübingen, Germany; 7 German Center for Diabetes Research (DZD E.V.), Neuherberg, Germany; 8 Chair of Epidemiology, LMU Munich, UNIKA-T Augsburg, Augsburg, Germany; 9 Institute for Biometrics and Epidemiology, German Diabetes Center, Düsseldorf, Germany; 10 KORA Study Centre, University Hospital of Augsburg, Augsburg, Germany; 11 Preventive Medicine and Epidemiology Section, Boston University School of Medicine and Framingham Heart Study, Framingham, MA USA; 12 Institute of Epidemiology, Kiel University, Kiel, Germany; 13 Leibniz Center for Diabetes Research at Heinrich Heine University, Düsseldorf, Germany

**Keywords:** Cardiac MRI, Cardiac function and structure, Risk factors, Cohort study

## Abstract

**Background:**

The association of longitudinal trajectories of cardiovascular risk factors with cardiovascular magnetic resonance (CMR)-measures of cardiac structure and function in the community is not well known. Therefore we aimed to relate risk factor levels from different examination cycles to CMR-measures of the left ventricle (LV) and right ventricle in a population-based cohort.

**Methods:**

We assessed conventional cardiovascular disease risk factors in 349 participants (143 women; aged 25–59 years) at three examination cycles (Exam 1 [baseline], at Exam 2 [7-years follow-up] and at Exam 3 [14-years follow-up]) of the KORA S4 cohort and related single-point measurements of individual risk factors and longitudinal trajectories of these risk factors to various CMR-measures obtained at Exam 3.

**Results:**

High levels of diastolic blood pressure, waist circumference, and LDL-cholesterol at the individual exams were associated with worse cardiac function and structure. Trajectory clusters representing higher levels of the individual risk factors were associated with worse cardiac function and structure compared to low risk trajectory clusters of individual risk factors. Multivariable (combining different risk factors) trajectory clusters were associated with different cardiac parameters in a graded fashion (e.g. decrease of LV stroke volume for middle risk cluster β = − 4.91 ml/m^2^, 95% CI − 7.89; − 1.94, p < 0.01 and high risk cluster β = − 7.00 ml/m^2^, 95% CI − 10.73; − 3.28, p < 0.001 compared to the low risk cluster). The multivariable longitudinal trajectory clusters added significantly to explain variation in CMR traits beyond the multivariable risk profile obtained at Exam 3.

**Conclusions:**

Cardiovascular disease risk factor levels, measured over a time period of 14 years, were associated with CMR-derived measures of cardiac structure and function. Longitudinal multivariable trajectory clusters explained a greater proportion of the inter-individual variation in cardiac traits than multiple risk factor assessed contemporaneous with the CMR exam.

## Introduction

The association of standard cardiovascular disease (CVD) risk factors with prevalent and incident CVD events [1–3] and with subclinical CVD traits, including parameters of left ventricular (LV) remodeling, is well established [4, 5]. In most studies, however, these risk factors were obtained only at one point in time. Data on longitudinal trajectories of risk factors and on the associations of these trajectories with subclinical and clinical CVD are limited [6]. In this context, it is not well known, whether information about cumulative risk factor burden over an extended period of time is more strongly related to clinical and subclinical CVD traits than measurements of CVD risk factors at one point in time (e. g. contemporaneous with the measurement of the CVD endpoint or trait) [7].

Cardiovascular magnetic resonance (CMR) is increasingly being used in population-based cohorts to measure in a more sensitive and specific way subclinical alterations of the cardiovascular system [8]. Few prior studies have related cardiac CMR-derived measures to established CVD risk factors, including systolic blood pressure (BP), body mass index (BMI) and total cholesterol, obtained at one point in time [9, 10]. These studies reported statistically significant associations, e. g. with LV end-diastolic volume, myocardial mass and ejection fraction [9, 10]. However, measurements of risk factors at one point in time do not adequately reflect long term exposure to risk factors and it is conceivable that cumulative exposure measures reflect cardiovascular risk better than single occasion measurements of one or multiple risk factors.

It is, therefore, of interest to evaluate the associations of CVD factors, measured repeatedly at different examination cycles, with CMR-parameters. In addition to the already established CMR markers mentioned above (e. g., myocardial mass), CMR provides a wide range of emerging measurements of cardiac structure and function including right ventricle (RV) function and epi- and pericardial fat that are available for further investigations [11, 12].

The secondary aims of the present study were to relate (i) 5 specific risk factor levels (systolic BP, diastolic BP, waist circumference, hemoglobin A1c (HbA1c), low density lipoprotein (LDL-C) from three individual exams (each about 7 years apart) as well as (ii) longitudinal trajectories of these individual CVD risk factors over 14 years to subclinical CMR-measures of LV volumes and mass, RV volumes and mass, and cardiac fat in a general population cohort (KORA project, conducted in southern Germany). The primary aim was to relate longitudinal clusters (over 14 year of follow-up) of *multiple* CVD risk factors to CMR traits independently of cross-sectional *multiple* risk factor clusters.

We hypothesized that (i) CVD risk factors levels measured contemporaneous with the CMR examination (Exam 3) are more strongly related to CMR traits than risk factor levels from more remote exams, and (ii) that trajectory clusters, which include information on the long-term trajectory of individual CVD risk factors are strongly related to CMR traits. Third, we hypothesized that trajectory clusters including multiple CVD risk factors and their change over time add more explanation to the inter-individual variation in CMR traits than multiple risk factors measured contemporaneous with the CMR exam (Exam 3).

## Methods

### Study sample

**Fig. 1:**
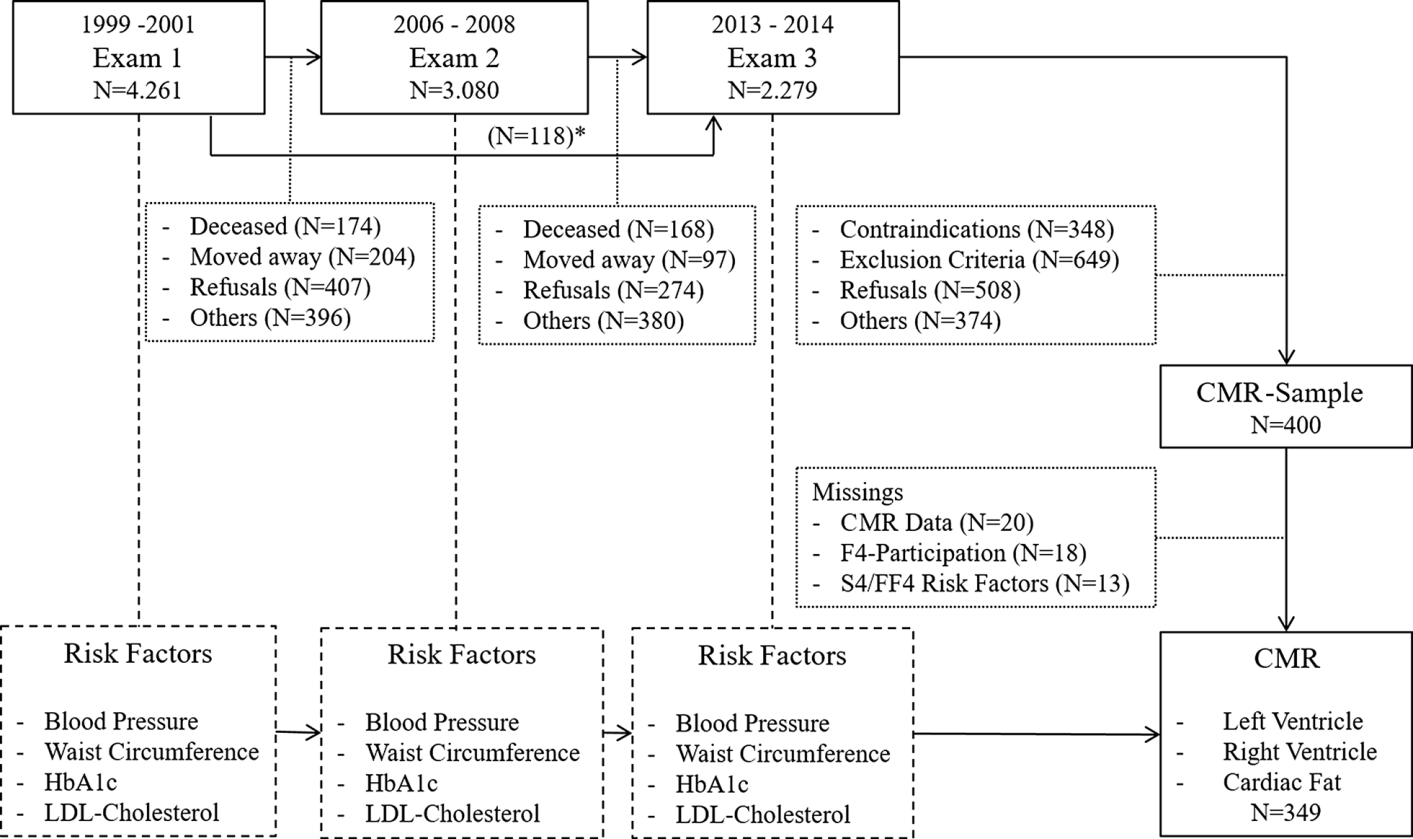
Flow chart of the KORA study sample (N = 349) and the cohort study design. (*N = 118 participants skipped Exam 2, but attended Exam 3)

The KORA S4 study (“Cooperative Health Research in the Region of Augsburg”) is a population-based cohort conducted in the city of Augsburg (Southern Germany) and two surrounding counties between 1999 and 2001 (baseline examination). Of all 4261 participants of the baseline examination cycle (Exam 1), 3080 individuals also participated in the 7-year follow-up examination (2006–2008; Exam 2) and 2279 subjects also participated in the 14-year follow-up examination (2013–2014; Exam 3) [13]. At Exam 3, a total of 400 participants without a history of stroke, myocardial infarction, and arterial vessel occlusion were examined by 3 T CMR in the KORA CMR sub-study that was planned with sufficient power for several analyses of subclinical cardiovascular disease differences in different risk groups [14]. Participants with missing data for cardiac imaging (n = 20), non-participation in Exam 2 (n = 18) and with missing data on risk factors and potential confounders at any of the Exams (n = 13) were excluded from the present analysis, yielding an analytical sample of 349 participants (143 women; aged 39 to 73 years) (Fig. 1).

### CMR examination and CMR-derived cardiac measurements

CMR examinations were performed on a 3 T system (Magnetom Skyra, Siemens Healthineers, Erlangen, Germany) using an 18 channel body array coil in combination with the table-mounted spine matrix coil [14]. Individuals were scanned in a supine position.

#### Left ventricle (LV)

Cine-balanced steady state free precession (bSSFP) sequences were evaluated semi-automatically using commercially available software (CVi42, Circle Cardiovascular Imaging, Calgary, Canada) by two alternative readers. Following automatic contour detection of the LV endocardium, all borders were corrected manually if necessary and according to current guidelines in order to avoid erroneous tracing of the myocardial border. Certification measurements prior to the analyses proved good reproducibility of the two readers with relative differences of less than 5% for LV volumes and ejection fraction [15]. For determination of filling and ejection rates, the time course of LV volume changes was quantified using associated gradients and time lags by using the dedicated in-house software. This software displays the LV volume versus time curve along with its derivative and estimates peak gradients during early, passive LV filling and late LV filling due to atrial contraction.

#### Right ventricle (RV)

Evaluation of RV parameters was performed using dedicated software (CVi42, Circle Cardiovascular Imaging Inc.) by one blinded, well-trained reader. Inter-reader (with other expert in cardiac imaging) and intra-reader-comparisons on 38 (9.5%) and 33 (8.3%) participants, respectively, were performed as part of the KORA quality control procedures. Deviations relative differences were < 5% for all parameters. End-diastole was defined as the phase with the biggest RV volume. End-systole was defined as the phase with the smallest RV volume. In the end-diastolic and end-systolic phase the contours of the RV lumen were manually delineated. The papillary muscles were included in the RV lumen. Slices from the apex up to the pulmonary valve were included in RV volume. The RV outflow tract was attributed to the RV [16]. For analysis, LV and RV volumes, as well as LV diastolic mass, were indexed to body surface area.

#### Cardiac fat

Analyses of the local fat compartments were conducted with a dedicated segmentation software (OsiriX Lite; Pixmeo SARL, Bernex, Switzerland). Epicardial fat was defined as the fat located inside the visceral layer of the pericardial sac in close proximity to the myocardium. Paracardial fat was defined as the fat compartment located outside of the pericardial sac. Together, these two fat compartments are summarized as pericardial fat. Epicardial and pericardial fat were assessed in Cine-bSSFP sequences (four chamber view) using only the diastolic phase and were segmented manually. Small non-fatty structures (e.g. coronary arteries) in the fat compartments were not segmented individually [17]. Data are given as an area in cm^2^.

For the main analyses, we investigated CMR parameters that were most strongly correlated with CVD risk factors in previous studies of this sample (LV: end-diastolic volume, stroke volume, diastolic myocardial mass, early diastolic filling rate; RV: end-diastolic volume, stroke volume; fat: epicardial, diastolic) [14, 16–18].

### Risk factor measurements

The aim of the study was to focus on 5 selected main cardiovascular risk factors (systolic BP, diastolic BP, waist circumference, HbA1c, LDL-C) that were modifiable, clinically measureable, lowly inter-correlated, and of a continuous measurement character. At each examination cycle, systolic and diastolic BP were measured three times at the right arm of seated participants after a minimum resting period of 5 min and a time interval of 3 min between measurements. An oscillometric digital BP monitor (HEM-705CP, Omron Corporation, Tokyo, Japan) and a cuff of appropriate size were used. The mean of the second and third BP measurements was used for the present analyses [19].

Waist circumference was measured with an inelastic multi-colored measuring tape (Fa Hoechstmass) in cm to the closest 0.1 cm. Waist circumference was measured at the level midway between the lower rib margin and the iliac crest while the participants breathed out gently.

Laboratory measurements including HbA1c, total cholesterol, high- and low-density lipoprotein cholesterol (HDL-C, LDL-C) were performed as described elsewhere [13]. Briefly, for the assessment of total cholesterol, LDL-C and HDL-C, enzymatic, photometric assays were used at the baseline examination and enzymatic, colorimetric Flex assays were used at Exam 2 and Exam 3. HbA1c was measured by a turbidimetric inhibition immunoassay at baseline (S4) and by cation-exchange high performance liquid chromatographic, photometric assays at Exam 2 and 3.

### Covariates

Further health-related variables were measured in a comparable fashion in all three KORA examination cycles by standardized interview and in a comprehensive physical examination. Participants were classified as never-smoker, ex-smoker or current smoker. Pack-years were calculated by multiplying the number of packs of cigarettes smoked per day by the number of years the participant has smoked. Participants were classified as being physically active if they did regular sports in summer and winter and were active for ≥ 1 h per week in at least one season or as physically inactive if they did less sports [20]. Alcohol intake was assessed using a validated recall method, calculating alcohol intake in grams per day from participants’ self-reported intake of beer, wine, sparkling wine or distilled spirits over the previous weekend and workday [21].

Diabetes was self-reported or defined by the use of glucose-lowering medication. Hypertension was defined as systolic BP ≥ 140 mmHg or diastolic BP ≥ 90 mmHg [22] or the use of antihypertensive medication under the awareness of having hypertension. Medication intake within the last seven days was recorded during a medical interview by computer-based software, and participants were also asked to bring their medication packages with them.

Weight and height were determined by Seca's measuring systems (Seca GmbH & Co, KG, Hamburg, Germany; [weight: Exam 1: SECA 709, Exam 2: SECA 709, Exam 3: SECA 635 or SECA 877 or SECA measuring station 285; height: Exam 1: SECA 221, Exam 2: SECA 242, Exam 3: SECA 242]) with either calibrated steelyards or digital scales allowing accurate measurements up to 0.1 kg and 0.1 cm, respectively. BMI was calculated as weight divided by squared height (kg/m^2^).

waist circumference was divided by hip circumference to get waist-hip-ratio (WHR) and by height to get waist-height-ratio (WHtR). Hip circumference was measured at the widest protrusion of the gluteal region between the superior border of the iliac crest and crotch. Body surface area (BSA) was calculated according to the Du Bois formula (BSA = 0.007184 × (height in cm)^0.725^ × (weight in kg)^0.425^) [23].

### Statistical analyses

For each KORA examination cycle, descriptive characteristics of participants are provided as means with standard deviations for continuous measurements and as absolute numbers and proportions for categorical measurements. Differences in participant characteristics between different KORA examination cycles were evaluated by one-way repeated measures ANOVA (continuous data) or Cochran's Q test (categorical data) [24]. MRI parameters from Exam 3 were also summarized as means with standard deviations.

#### Association of risk factor levels from individual exams with CMR traits

Associations of five risk factors from three individual exams (each risk factor at each exam considered separately as independent variables) with different CMR-derived parameters of cardiac function and structure (dependent variables) were assessed by multivariable linear regression models (providing β-coefficients with 95% confidence intervals). The following risk factors were used as exposure variables: systolic BP, diastolic BP, waist circumference, HbA1c and LDL-C. The models included the following potential confounders (obtained at each exam): age, sex, smoking status, alcohol consumption, physical activity, antihypertensive medication, lipid-lowering medication, and glucose-lowering medication. The explained outcome variance for each model was measured by the Goodness-Of-Fit statistic R^2^.

#### Derivation of risk factor trajectory clusters

Longitudinal risk factor trajectory clusters were derived by non-parametric k-means clustering [25, 26]. Measurements of 5 risk factors (systolic and diastolic BP, waist circumference, HbA1c and LDL-cholesterol), each obtained at the three examination cycles (Exams 1–3) were used as input values. Individuals with a high similarity regarding mean values at Exam 1–3 and regarding change in risk factor levels between exams were clustered together, whereas participants with lower similarity in risk factor levels and trajectories were assigned to different clusters [27]. Similarity was determined by the Euclidean distance—the shorter the Euclidean distance between two subjects, the more similar the subjects are.

For individual risk factor trajectories, only the values of a single risk factor at Exams 1–3 were considered. For multivariable risk factor trajectories, the values of all five risk factors were considered conjointly: In the multivariate k-means algorithm each subject is represented as a data matrix with columns denoting the time points of measurements and rows denoting the different risk factors. Values are then subsumed by calculating the distance between the column vectors and applying a mathematical norm function on the resulting vector of distances [25, 26].

As there is no established criterion to determine the “correct” number k of clusters for a k-means algorithm, a combination of domain knowledge and heuristic criteria was used. To obtain informative clusters, the possible number of clusters was pre-set to be > 2 but < 8. Of the resulting set of possible number of clusters, all numbers were tested on the multivariable risk factor trajectories. The final number was determined by maximizing the Calinski-Harabasz Index [28], which is essentially given by the ratio of the variance between clusters to the variance within clusters. The index is, therefore, maximized for clusters which are compact and well separated from each other. Based on this criterion, the optimal number of clusters was three, representing low, middle and high cumulative exposure to the respective risk factors over 14 years. Consequently, k = 3 was also used for the individual risk factor trajectories.

#### Association of individual risk factor trajectories with CMR traits

CVD risk factor trajectories for individual risk factors were displayed graphically by plotting the mean risk factor level in each of the three clusters (low, middle and high exposure) at each of the Exams. The associations of the trajectory clusters with the different CMR outcomes were analyzed by multiple linear regression, adjusted for the potential confounders as detailed above. Models including trajectory clusters of individual risk factors were additionally adjusted for the other remaining 4 CVD risk factors. Thus, the trajectory cluster for systolic BP was adjusted for the confounders listed above and in addition for diastolic BP, HbA1c, waist circumference and LDL-C (all measured at Exam 3, contemporaneous with the CMR).

#### Association of multiple risk factor trajectories with CMR traits

To quantify the associations of the multivariable risk clusters with different CMR outcomes, adjusted predicted means of the different CMR traits were stratified by clusters (reflecting low, moderate and high cumulative exposure to multiple CVD risk factors over 14 years) and were displayed graphically using the estimates of multivariable linear regression models. Also these analyses were adjusted for the potential confounders as detailed above.

To investigate the incremental value of *longitudinal* information from multiple risk factor beyond multiple risk factor information from Exam 3 (contemporaneous with the CMR; cross-sectional) for the explanation of CMR outcomes, a likelihood ratio (LR) test was applied, comparing a model with multivariable risk factors clusters from Exam 3 only (cross-sectional) to a model which includes longitudinal multivariable risk factor trajectory cluster (over 14 years) and the cross-sectional multivariable risk factor cluster from Exam 3.

A two-sided p-value of < 0.05 was considered statistically significant. For the final main analyses including eight LR-tests we adjusted the level of significance by p < 0.05/8 (= 0.0063). Statistical analyses were performed using Stata 16.1 (Stata Corporation, College Station, Texas, USA) and R 3.4.1 (R Core Team, Vienna, Austria).

## Results

Over the 14-year period from Exam 1 to Exam 3, we observed an increase in hypertension prevalence (29–33%), and in mean levels of HbA1c (5.48–5.56%), waist circumference (90.6–98.4 cm) and LDL-cholesterol (133.0–138.9 mg/dl; Table 1). Furthermore, mean systolic and diastolic BP decreased slightly from Exam 1 to Exam 3, whereas the intake of antihypertensive medication increased substantially from 7.7 to 24.9%, which might contribute to the decrease in mean systolic BP over time, since it is well established that systolic BP usually increases with age [29]. The prevalence of smokers dropped from 27% (Exam 1) to 20% (Exam 3), whereas the proportion of physically active participants increased from 51% (Exam 1) to 61% (Exam 3; Table 1).

**Table 1:** Cardiovascular disease risk factors at Exam 1, Exam 2 and Exam 3 (N = 349)

	Exam 1 (1999–2001)	Exam 2 (2006–2008)	Exam 3 (2013–2014)	p value*
Age (years)	42.5 (± 9.2)	49.5 (± 9.2)	56.5 (± 9.2)	–
Males	206 (59.0%)	206 (59.0%)	206 (59.0%)	–
Systolic BP (mmHg)	126.2 (± 16.1)	121.1 (± 16.2)	120.2 (± 16.1)	< 0.001
Diastolic BP (mmHg)	81.6 (± 10.3)	76.6 (± 9.4)	75.2 (± 9.8)	< 0.001
Hypertension	101 (28.9%)	91 (26.1%)	115 (33.0%)	0.009
Weight (kg)	78.8 (± 13.6)	81.4 (± 15.2)	82.8 (± 16.2)	< 0.001
Height (cm)	171.9 (± 9.6)	172.5 (± 9.8)	171.9 (± 9.9)	< 0.001
Body mass index (kg/m^2^)	26.6 (± 3.8)	27.3 (± 4.2)	28.0 (± 4.7)	< 0.001
Waist circumference (cm)	90.6 (± 11.6)	93.8 (± 12.9)	98.4 (± 13.8)	< 0.001
Hip circumference (cm)	104.4 (± 7.1)	106.0 (± 7.9)	106.7 (± 8.8)	< 0.001
Waist-hip-ratio	0.87 (± 0.08)	0.88 (± 0.08)	0.92 (± 0.09)	< 0.001
Waist-height-ratio	0.53 (± 0.06)	0.54 (± 0.07)	0.57 (± 0.08)	< 0.001
Body surface area (m^2^)	1.91 (± 0.2)	1.95 (± 0.21)	1.95 (± 0.22)	< 0.001
Smoking status				
Never-smoker	131 (37.5%)	131 (37.5%)	131 (37.5%)	–
Ex-smoker	124 (35.5%)	140 (40.1%)	148 (42.4%)	0.001
Current smoker	94 (26.9%)	78 (22.4%)	70 (20.1%)	0.001
Pack-years	10.8 (± 16.6)	12.3 (± 18.4)	13.1 (± 19.3)	< 0.001
Alcohol consumption (g/day)	19.2 (± 25.9)	18.8 (± 24.4)	19.1 (± 24.4)	0.734
Physically active	178 (51%)	205 (58.7%)	212 (60.7%)	0.002
Diabetes mellitus	4 (1.2%)	13 (3.7%)	28 (8.0%)	0.003
HbA1c (%)	5.48 (± 0.47)	5.48 (± 0.53)	5.56 (± 0.71)	< 0.001
Total cholesterol (mg/dl)	223.0 (± 40.6)	215.5 (± 37.4)	217.3 (± 36.4)	< 0.001
HDL-C (mg/dl)	56.2 (± 16.6)	54.1 (± 13.7)	62.5 (± 17.4)	< 0.001
LDL-C (mg/dl)	133.4 (± 39.1)	137.7 (± 33.7)	138.9 (± 32.9)	0.011
Antihypertensive medication	27 (7.7%)	53 (15.2%)	87 (24.9%)	0.034
Lipid-lowering medication	7 (2%)	23 (6.6%)	36 (10.3%)	0.003
Glucose-lowering medication	4 (1.2%)	9 (2.6%)	26 (7.5%)	0.003
Statin medication	7 (2%)	20 (5.7%)	36 (10.3%)	0.003

Data are given as number (percentage) or mean (± standard deviation)

*BP* blood pressure, *HbA1c* hemoglobin A1c, *HDL-C* high-density-lipoprotein cholesterol, *LDL-C* low-density-lipoprotein cholesterol

^*^One-way repeated measures ANOVA (continuous data) or Cochran's Q test (categorical data)

Characteristics of CMR-derived parameters of cardiac function and structure, measured at Exam 3 are summarized in Table 2.

**Table 2:** Cardiovascular magnetic resonance (CMR) traits of cardiac function and structure, obtained at Exam 3, stratified by sex (N = 349)

MRI traits	Exam 3 (2013–2014)	
	Women (n = 143)	Men (n = 206)
Left ventricle		
End-diastolic volume (ml)	116.1 (± 26.7)	139.0 (± 34.2)
End-systolic volume (ml)	34.1 (± 15.0)	45.6 (± 19.0)
Stroke volume (ml)	82.1 (± 17.4)	93.4 (± 21.6)
Ejection fraction (%)	71.3 (± 7.0)	67.9 (± 8.1)
Myocardial mass, diastolic (g)	114.6 (± 25.9)	159.3 (± 27.7)
Myocardial mass, systolic (g)	113.7 (± 26.7)	164.5 (± 30.8)
Early diastolic filling rate (ml/s)	224.4 (± 105.8)	227.2 (± 120.9)
Peak ejection rate (ml/s)	328.7 (± 106.4)	373 (± 146.8)
Right ventricle		
End-diastolic volume (ml)	143.1 (± 30)	181.7 (± 38.5)
End-systolic volume (ml)	63.6 (± 18.4)	90.7 (± 24.4)
Stroke volume (ml)	79.5 (± 16.9)	91.1 (± 20.2)
Ejection fraction (%)	55.8 (± 6.4)	50.4 (± 6.6)
Cardiac fat		
Epicardial fat, diastolic (cm^2^)	6.6 (± 3.5)	9.2 (± 4.3)
Pericardial fat, diastolic (cm^2^)	18.6 (± 9.8)	33.0 (± 16)

Data are given as mean (± standard deviation)

### Individual cardiovascular disease risk factors from Exam 1–3 and cardiac remodeling traits and fat

The associations of 5 main CVD risk factors (systolic and diastolic BP, waist circumference, HbA1c and LDL-C) from three examination cycles with various CMR outcome variables are displayed in Additional file 1: Table S1. In essence, diastolic BP, waist circumference and LDL-C displayed consistent inverse associations with end-diastolic volumes and stroke volumes of the LV and the RV, respectively, across the three examination cycles. The strength of association was slightly stronger for BP traits and waist circumference from Exam 2 and 3 (contemporaneous with the CMR) than with the risk factors from Exam 1. WC and, to a certain extent also diastolic BP, were positively associated with epicardial and/or pericardial fat (Additional file 1: Table S1).

Systolic BP was most consistently associated with LV stroke volume and myocardial mass. These associations were slightly stronger for systolic BP measurements obtained at Exam 2 and 3, as compared to systolic BP obtained at Exam 1 (Additional file 1: Table S1).

By contrast, HbA1c did not provide evidence for a consistent association with CMR traits of cardiac structure and function or with cardial fat, even though we observed a statistically significant association of HbA1c from the Exam 3 with stroke volume and myocardial mass. However, these associations could not be confirmed with HbA1c levels assessed at the Exam 1 and 2 (Additional file 1: Table S1).

In aggregate, the risk factor profiles from the Exam 3 (contemporaneous with the CMR measurements) explained slightly more variation in the different CMR outcomes than the risk factor levels obtained at baseline or at Exam 2: R^2^-values for the regression models increased slightly but consistently for all cardiac function and structure parameters from the first to the third examination cycle (Additional file 1: Table S1).

### Individual Risk factor trajectory clusters and cardiac remodeling

**Fig. 2:**
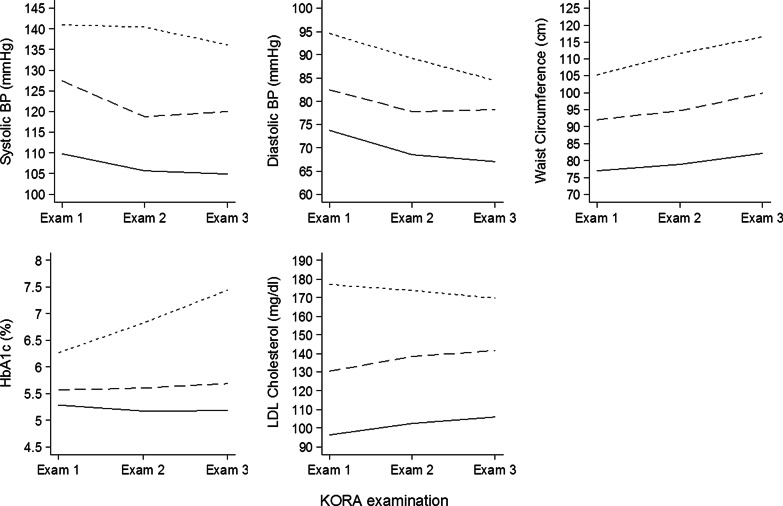
Change in mean levels for cardiovascular disease (CVD) risk factors from Exam 1 (baseline) to Exam 2 (7-year follow-up) and Exam 3 (14-year follow-up) for the three clusters derived from each individual CVD risk factor, respectively (grouping individuals with similar mean levels and trajectories (solid line = low level cluster, long dashed line = middle level cluster and short dashed line = high level cluster) (N = 349)

For each risk factor, we defined three trajectory clusters based on mean risk factor levels and the change of the risk factors over time (Fig. 2). Individuals with similar mean values and comparable longitudinal trajectories were grouped together, as detailed in the methods section. In the high and low-level clusters, mean values for systolic and diastolic BP decreased whereas mean values for waist circumference increased over the 14 year follow-up period (Fig. 2). Mean HbA1c values increased substantially in the high level cluster (reflecting a higher proportion of incident diabetes cases; n = 15), but remained essentially unchanged in the middle (incident diabetes cases n = 9) and low-level cluster (n = 0). Mean LDL-C levels slightly decreased in the high-level cluster, whereas LDL-C levels in the middle and low-level cluster slightly increased.

The association of the different individual CVD risk factor trajectory clusters with CMR measures is displayed in Table 3. Consistent with the associations of individual risk factors measurements (Additional file 1: Table S1), the clusters representing high cumulative exposure (high mean levels) of waist circumference, diastolic BP and LDL-C displayed the most consistent evidence for association with end-diastolic diameters and stroke volumes of the LV and the RV (inverse associations) as well as with epi- and pericardial fat (positive associations for waist circumference and diastolic BP) (Table 3). For waist circumference, the mid-level trajectory cluster displayed a comparable pattern of association (same directionality, lower strength of association) as the high level trajectory (Table 3).

**Table 3:** Associations of trajectory clusters for individual CVD risk factors (representing low, medium and high cumulative exposure to this risk factor over 14 years) with CMR derived measures of cardiac structure and function

CV risk factor clusters^a^	N	Left ventricle				Right ventricle		Cardiac fat	
		End-diastolic volume^b^	Stroke volume^b^	Myocardial mass, diastolic^b^	Early diastolic filling rate	End-diastolic volume^b^	Stroke volume^b^	Epicardial fat, diastolic	Pericardial fat, diastolic
	349	β (95% CI)	β (95% CI)	β (95% CI)	β (95% CI)	β (95% CI)	β (95% CI)	β (95% CI)	β (95% CI)
Systolic BP (low)	101	Ref	Ref	Ref	Ref	Ref	Ref	Ref	Ref
(middle)	149	0.03 (− 4.13; 4.19)	1.00 (− 1.58; 3.57)	2.13 (− 1.32; 5.58)	− 18.47 (− 48.99; 12.04)	− 0.26 (− 4.9; 4.37)	0.97 (− 1.56; 3.5)	− 0.40 (− 1.44; 0.65)	− 2.68 (− 6.10; 0.75)
(high)	99	3.23 (− 2.48; 8.93)	2.46(− 1.07; 6.00)	7.42** (2.69; 12.16)	− 31.73 (− 73.58; 10.13)	1.73 (− 4.72; 8.19)	2.12 (− 1.40; 5.64)	0.02 (− 1.41; 1.45)	0.09 (− 4.58; 4.76)
Diastolic BP (low)	135	Ref	Ref	Ref	Ref	Ref	Ref	Ref	Ref
(middle)	142	− 2.76 (− 6.83; 1.3)	− 1.42 (− 3.93; 1.08)	− 1.29 (− 4.64; 2.05)	− 24.42 (− 54.13; 5.29)	− 2.01 (− 6.55; 2.52)	− 1.06 (− 3.53; 1.40)	0.11 (− 0.90; 1.11)	− 0.80 (− 4.13; 2.52)
(high)	72	− 4.63 (− 10.32; 1.06)	− 4.61* (− 8.12; − 1.10)	− 2.53 (− 7.21; 2.16)	− 54.49* (− 96.09; − 12.89)	− 5.77 (− 12.05; 0.51)	− 4.31* (− 7.72; − 0.90)	2.12** (0.71; 3.52)	5.02* (0.39; 9.65)
Waist circumference (low)	97	Ref	Ref	Ref	Ref	Ref	Ref	Ref	Ref
(middle)	180	− 4.31* (− 8.53; − 0.09)	− 3.18* (− 5.79; − 0.56)	2.23 (− 1.18; 5.65)	− 10.68 (− 41.49; 20.12)	− 6.59** (− 11.39; − 1.79)	− 3.05* (− 5.66; − 0.43)	2.21*** (1.11; 3.31)	6.42** (2.68; 10.16)
(high)	72	− 8.9** (− 14.36; − 3.45)	− 5.47** (− 8.86; − 2.09)	1.90 (− 2.51; 6.31)	− 22.5(− 62.3; 17.29)	− 10.62** (− 16.81; − 4.44)	− 4.54** (− 7.9; − 1.18)	4.29*** (2.89; 5.70)	15.31*** (10.52; 20.11)
HbA1c (low)	159	Ref	Ref	Ref	Ref	Ref	Ref	Ref	Ref
(middle)	169	− 1.23 (− 4.53; 2.08)	− 1.26 (− 3.30; 0.79)	0.35 (− 2.40; 3.09)	− 0.21 (− 24.52; 24.11)	− 0.25 (− 3.91; 3.41)	− 0.49 (− 2.49; 1.51)	− 0.45 (− 1.28; 0.39)	− 0.99 (− 3.75; 1.76)
(high)	21	6.24 (− 4.98; 17.47)	1.31 (− 5.65; 8.26)	7.92 (− 1.41; 17.25)	58.2 (− 24.41; 140.82)	7.05 (− 5.68; 19.78)	2.22 (− 4.73; 9.17)	− 3.28* (− 6.13; − 0.42)	− 8.44 (− 17.86; 0.97)
LDL-C (low)	103	Ref	Ref	Ref	Ref	Ref	Ref	Ref	Ref
(middle)	149	− 1.41 (− 5.06; 2.24)	− 1.19 (− 3.43; 1.05)	− 1.91 (− 4.91; 1.1)	− 1.60 (− 28.45; 25.26)	1.15 (− 2.88; 5.18)	0.19 (− 2.00; 2.38)	− 0.38 (− 1.32; 0.55)	− 0.19 (− 3.28; 2.89)
(high)	97	− 6.75** (− 10.96; − 2.54)	− 5.16*** (− 7.74; − 2.58)	− 2.77 (− 6.23; 0.69)	− 33.48* (− 64.41; − 2.55)	− 6.14* (− 10.82; − 1.46)	− 3.54* (− 6.09; − 1.00)	0.05 (− 1.01; 1.12)	1.22 (− 2.29; 4.73)

Data are β coefficients (with 95% confidence interval; indicating change in outcome variable between reference cluster and risk factor cluster) from multivariable linear regression models adjusted for the other risk factors respectively and for age, sex, smoking status, alcohol consumption, physical activity, antihypertensive medication, lipid-lowering medication, glucose-lowering medication measured in Exam 3

*Ref.* reference cluster

***p < 0.001; **p < 0.01; *p < 0.05

^a^Clusters were calculated by k-means clustering

^b^Indexed to body surface area

### Multivariable CVD risk factor trajectory clusters and cardiac remodeling

**Fig. 3:**
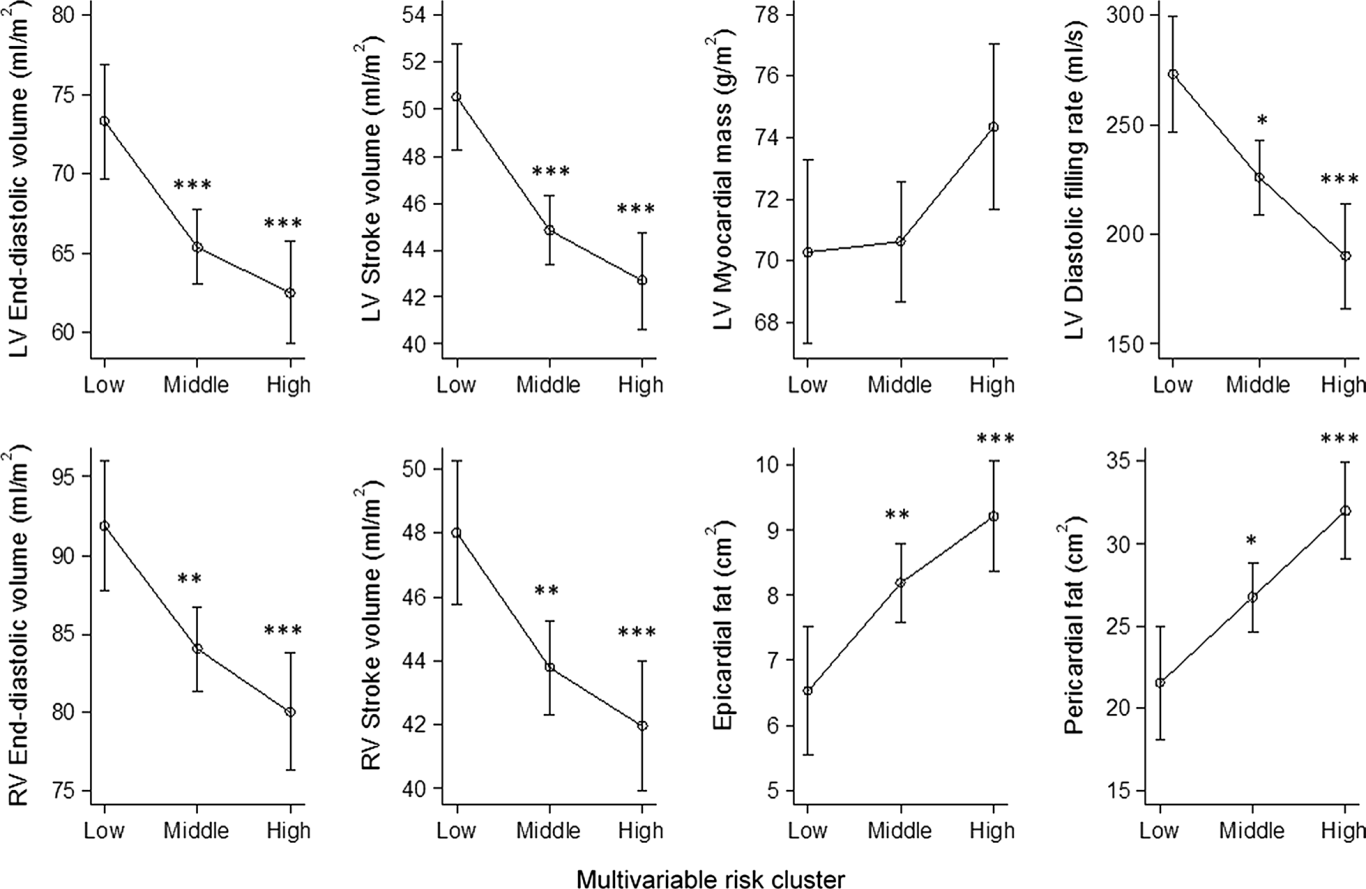
Associations of multivariable risk clusters (including information from the following risk factors: systolic and diastolic blood pressure (BP), waist circumference, HbA1c, and LDL-C) with CMR-traits of cardiac structure and function (N = 349). Displayed are predicted mean values for the CMR traits, stratified by multivariable risk cluster (representing low, medium and high cumulative risk factor exposure). The analyses were adjusted for age, sex, smoking status, alcohol consumption, physical activity, antihypertensive medication, lipid-lowering medication and glucose-lowering medication measured at Exam 3 (contemporaneous with the CMR examination). The lines connecting the dots from low to medium and high are for visual aid only. (***p < 0.001; **p < 0.01; *p < 0.05, reference = low-level cluster). LV = left ventricle; RV = right ventricle

We also estimated multivariable trajectory clusters, taking into account longitudinal information from all five CVD risk factors (systolic and diastolic BP, waist circumference, HbA1c, LDL-C). These multivariable risk factor trajectories were clearly and consistently associated with nearly all CMR traits, except for LV mass (which was specifically related to high systolic BP), and with epicardial and pericardial fat. Figure 3 displays the adjusted means of all CMR traits according to the three multivariable risk factor trajectory clusters. In essence, we observed a graded relation between the multivariable trajectory clusters and most CMR traits in the sense that higher exposure to multiple risk factors over time (the middle risk and the high risk trajectories) was associated with lower end-diastolic volumes of the LV and RV, lower stroke volumes of the LV and RV, lower LV diastolic filling rate, as well as with higher levels of epicardial and pericardial fat.

Model A in Table 4 denotes the cross-sectional association of multivariable risk factor clusters from Exam 3 with CMR traits. Once longitudinal information from the multivariable risk factor trajectory clusters is added to the cross-sectional multivariable risk clusters from Exam 3 (Table 4; Model B), the model fit improved [LR tests were statistically significant (Table 4)], and the risk factor clusters from Exam 3 only were no longer statistically significant whereas the longitudinal multivariable risk factor trajectory clusters were statistically significantly associated with various CMR traits (Table 4; Model B).

**Table 4:** Cross-sectional association of multivariable CVD risk factor clusters from Exam 3 (representing low, medium and high exposure to 5 CVD risk factors at this exam) with CMR derived measures of cardiac structure and function (Model A); and a combined model displaying the association of multivariable risk factor clusters of Exam 3 (contemporaneous with the CMR) AND of the longitudinal (over 14-years) multivariable risk factor clusters with MR derived measures of cardiac structure and function (Model B)

CV Risk factors	N	Left ventricle				Right ventricle		Cardiac fat	
		End-diastolic volume^b^	Stroke volume^b^	Myocardial mass, diastolic^b^	Early diastolic filling rate	End-diastolic volume^b^	Stroke volume^b^	Epicardial fat, diastolic	Pericardial fat, diastolic
	349	β (95% CI)	β (95% CI)	β (95% CI)	β (95% CI)	β (95% CI)	β (95% CI)	β (95% CI)	β (95% CI)
Model A									
Cross-sectional multivariable risk factor clusters^a^									
Low	104	Ref	Ref	Ref	Ref	Ref	Ref	Ref	Ref
Middle	164	− 2.77 (− 6.58; 1.05)	− 2.19 (− 4.6; 0.23)	− 0.50 (− 3.63; 2.63)	− 23.07 (− 50.8; 4.65)	− 2.23 (− 6.56; 2.09)	− 1.56 (− 3.91; 0.79)	− 0.35 (− 1.37; 0.67)	0.76 (− 2.78; 4.31)
High	81	− 6.51** (− **11.09; **− **1.94)**	− 5.01** (− **7.91; **− **2.11)**	1.64 (− 2.12; 5.4)	− 51.44** (− **84.7; **− **18.17)**	− 7.94** (− **13.16; **− **2.71)**	− 4.04** (− **6.87; **− **1.20)**	0.20 (− 1.03; 1.42)	2.84 (− 1.39; 7.08)
Model B									
Cross-sectional multivariable risk factor clusters^a^									
Low	104	Ref	Ref	Ref	Ref	Ref	Ref	Ref	Ref
Middle	164	− 0.05 (− 4.14; 4.03)	− 0.30 (− 2.88; 2.28)	− 1.04 (− 4.43; 2.35)	− 6.46 (− 36.19; 23.28)	0.44 (− 4.2; 5.07)	− 0.18 (− 2.7; 2.34)	− 1.21* (− **2.30; **− **0.12)**	− 1.74 (− 5.52; 2.05)
High	81	− 2.95 (− 7.93; 2.02)	− 2.52 (− 5.66; 0.61)	0.89 (− 3.23; 5.02)	− 29.47 (− 65.65; 6.72)	− 4.42 (− 10.08; 1.25)	− 2.22 (− 5.3; 0.86)	− 0.91 (− 2.22; 0.40)	− 0.41 (− 4.97; 4.14)
Longitudinal multivariable risk factor trajectory clusters^c^									
Low	83	Ref	Ref	Ref	Ref	Ref	Ref	Ref	Ref
Middle	159	− 7.11** (− **11.83; **− **2.39)**	− 4.91** (− **7.89; **− **1.94)**	0.29 (− 3.63; 4.21)	− 37.71* (− **72.05; **− **3.37)**	− 6.72* (− **12.09; **− **1.35)**	− 3.56* (− **6.48; **− **0.64)**	2.20** (0.93; 3.46)	5.77* (1.39; 10.16)
High	107	− 9.93** (− **15.83; **− **4.04)**	− 7.00*** (− **10.73; **− **3.28)**	4.21 (− 0.68; 9.1)	− 72.32** (− **115.22; **− **29.42)**	− 10.76** (− **17.44; **− **4.09)**	− 5.41** (− **9.04; **− **1.77)**	3.28*** (1.72; 4.84)	11.05*** (5.62; 16.47)
LR-test^a^		p = 0.003	p < 0.001	p = 0.068	p = 0.003	p = 0.006	p = 0.011	p < 0.001	p < 0.001

Data are β-coefficients (with 95% confidence interval; indicating change in outcome variable between reference cluster and risk factor cluster) from multivariable linear regression models adjusted for age, sex, smoking status, alcohol consumption, physical activity, antihypertensive medication, lipid-lowering medication, glucose-lowering medication measured in Exam 3

*Ref.* reference cluster

***p < 0.001; **p < 0.01; *p < 0.05

^a^Multivariable clusters included the risk factors systolic BP, diastolic BP, WC, HbA1c and LDL-C and were calculated by k-means clustering

^b^Indexed to body surface area

^c^Likelihood-Ratio-test for adding the longitudinal multivariable risk factor trajectory clusters to the cross-sectional multivariable risk factor clusters (Model B)

Thus, the (longitudinal) multivariable trajectory clusters added significant information in explaining variation of the various CMR traits beyond the multivariable risk profile obtained at Exam 3 (contemporaneous with the CMR exam, Table 4, all p values ≤ 0.011, except for myocardial mass). Results of further risk factor clusters and CMR parameters are available in Additional file 1: Tables S2, S3 and S4, respectively. Sensitivity analyses with adjustment for covariates from Exam 1 (Additional file 1: Table S5) did not reveal substantially different results.

## Discussion

In a general population cohort, we investigated the associations of individual CVD risk factors from three examination cycles (covering a period of 14 years from Exam 1 to Exam 3) with CMR measures obtained at Exam 3. We also assessed how strong longitudinal trajectory clusters of individuals risk factors were associated with CMR measures and whether longitudinal trajectory clusters of multiple CVD risk factors add information to risk factor measurements obtained concurrent to the CMR with respect to explaining variation in CMR measures.

### Principal observations

The results can be summarized as follows: First, individual measurements of diastolic BP, waist circumference and LDL-C were consistently, inversely associated with LV and RV stroke volumes and end-diastolic volumes. The association of these risk factors with CMR traits was slightly stronger for risk factors from Exam 2 and 3 (closer to the CMR exam conducted at Exam 3), compared to Exam 1. Second, mainly waist circumference, but also diastolic BP were associated with cardiac fat depots. Third, systolic BP was most significantly associated with myocardial mass, and—to some extent—with LV stroke volume. Fourth, HbA1c did not display a consistent pattern of association with CMR traits or epi- and pericardial fat. Fifth, long-term exposure to high levels of diastolic BP, waist circumference and LDL-C (high risk factor levels trajectories) displayed statistically significant associations with end-diastolic volumes and stroke volumes of both ventricles. Sixth, long-term exposure to high and medium levels of several risk factors (multivariable trajectory clusters) displayed strong and consistent associations with multiple CMR traits and fat depots and added additional information for the explanation of CMR outcomes beyond multivariable risk measurement from Exam 3 (concurrent to the CMR exam).

### Association of individual risk factor measurements with cardiac remodeling

We observed consistent associations of diastolic BP, waist circumference and LDL-C with LV and RV end-diastolic and stroke volume. While the associations of these CVD risk factors from Exam 2 and 3 were slightly stronger than with risk factors measured at Exam 1, defined risk factors from Exam 1 also displayed significant associations with CMR traits obtained 14 years later at Exam 3. In essence, Exam 1 BP traits and LDL-C were associated with LV mass and stroke volume and Exam 1 waist circumference was related to RV parameters and to cardiac fat depots.

The inverse association of diastolic BP, waist circumference and LDL-C with filling rates, end-diastolic volume and stroke volume could be consistent with these risk factors contributing to smaller stiffer ventricles which are potential precursors of heart failure with preserved ejection fraction later in life [30].

Prior studies provided cross-sectional evidence for associations of standard cardiovascular risk factors with subclinical cardiac remodeling [9–11, 31]. In a sample of the UK Biobank, the impact of several modifiable risk factors on CMR-measured subclinical alterations of all four cardiac chambers was analyzed [10]. Similarly to our study, higher systolic BP was related to higher LV mass and volumes while higher diastolic BP and higher cholesterol levels were related to lower LV mass and volumes.

The association of systolic BP with LV mass was a prominent results in our analyses and agrees well with another study [31]. In fact, high LV mass is considered an integral measure of long-time exposure to high BP and it has previously been demonstrated in data from the Framingham Heart Study that systolic BP is an important correlate of LV mass progression over 4 years as well as over the adult life course [31]. However, a bit surprisingly, systolic BP was not significantly associated with other CMR traits which is in part in contrast with prior observations [9–11].

In addition, higher BMI was previously associated with higher LV mass and volumes not indexed by BSA [10] as compared to our study that demonstrated an inverse association of waist circumference with LV mass and volumes. Our results regarding the associations of BP and cholesterol levels with cardiac parameters are also in line with results of a CMR examination of the RV conducted within the community-based MESA study [11]. In the latter study, total cholesterol levels were inversely associated with mass and end-diastolic volume of the LV and RV while HDL-C was positively associated with end-diastolic volume in both ventricles. In contrast, Framingham Heart Study investigators did not reveal independent associations between lipid concentrations and echocardiographic measurements of LV structure in individuals free of CVD [32].

While clinically overt diabetes is associated with an increased risk for adverse cardiac remodeling [10, 11] and incident heart failure [33], it is less clear, whether HbA1c level in a general population sample (including many non-diabetic individuals) confer an increased risk for adverse cardiac remodeling. Analyses in the likewise community-based SHIP study displayed a lack of association of HbA1c levels with LV mass index and fractional shortening, key ultrasonographic measures of cardiac structure and function, respectively [34].

### Antecedent risk factor levels and CVD

Data on the association of antecedent risk factor levels with clinical CVD events have been published in different cohort studies [35–37]. Lee and colleagues investigated in the Framingham Heart Study the associations of remote (− 30 years), recent (− 20 years) and current (− 10 years) BP and BMI levels with incident heart failure and revealed that all three measurements of systolic BP, pulse pressure, and BMI were associated with incident heart failure independently of other covariates [36]. This study could not detect a significant impact of current, recent and remote diastolic BP on heart failure risk. Framingham Heart Study investigators have also reported a positive association between antecedent levels of systolic BP, diastolic BP and pulse pressure with the 10-year risk of overall CVD events, including death, myocardial infarction, stroke, claudication, heart failure and others in subgroups of sex and age [37]. A more recent study reported that antecedent systolic BP was a stronger predictor of the 20-year risk of CVD events than current systolic BP, whereas—in the same sample—, antecedent hypertension was not a significant predictor for incident CVD [35]. A further study observed the relation between lipid concentrations and incident heart failure over a long time period (mean = 26 years) and revealed a higher heart failure risk for higher non-HDL-C levels and a lower heart failure risk for higher HDL-C levels [38].

### Risk factor trajectories and CVD

This is the first study to assess associations of repeated risk factor measurements and their trajectories over a 14 year time period with CMR-based measures of cardiac structure and function as well as peri- and epicardial fat. The evidence linking risk factor trajectories to subclinical CVD is not yet available in contrast to the evidence for overt CVD.

In a community-based sample of elderly (≥ 65 years) individuals, BP measurements were recorded at least 4 times over a period of 7 years. The relation of BP trajectory clusters (cluster 1: increasing systolic and diastolic BP from low levels; cluster 2: constant systolic BP and decreasing diastolic BP; cluster 3: decreasing systolic and diastolic BP from high levels) to all-cause mortality, incident CVD (fatal and nonfatal cerebrovascular accidents and myocardial infarctions) and incident heart failure resulted in cluster 3 to be exposed to highest risk for all three outcomes compared to cluster 1 [39]. Attard and colleagues reported 10 different longitudinal BMI trajectories from adolescence to adulthood (mean age 17–29 years) and their association with CVD risk [40] over a period of 12 years. These different BMI trajectories were associated variably with higher risk of diabetes, hypertension and inflammation (as main risk factors for cardiac remodeling) [41] compared to the referent cluster of constant low BMI over time in both, in women and men.

A further study compared metabolic and biochemical risk factor trajectories between a group of incident CVD cases and an age- and sex-matched control group over a time period up to 15–20 years and revealed that systolic BP and waist circumference trajectories were more unfavorable in the CVD group compared to the control group [42]. However, trajectories of BMI, diastolic BP, total and HDL-C levels were not different between both groups of that latter study. This is partly in line with the results from our analysis, where we observed that only some single risk factor trajectory clusters (diastolic BP, waist circumference, LDL-C) were associated with selected cardiac parameters.

However, in our sample the multivariable clusters incorporating all risk factors simultaneously were significantly associated to all cardiac outcomes except LV mass, which underscores the importance of taking the conjoint evolution of risk factors into account.

### Strengths and limitations

Strengths of our study include the well-characterized sub-sample of the population-based KORA study, a large prospective cohort study with detailed and highly standardized measurements of a wide range of established cardiovascular risk factors and other phenotypes. Furthermore, we used advanced CMR techniques and quality assured reading procedures to characterize subclinical parameters of cardiac structure and function. To assess the associations between risk factors and CMR parameters we used not only risk factor measurements of the same examination cycle as the CMR examination (Exam 3) but also the examination cycles 7-years and 14-years prior the CMR examination.

The following limitations merit consideration. We conducted the CMR examination only at Exam 3. Thus, we were not able to investigate the associations of risk factor levels from prior exams or risk factor trajectories with changes in subclinical CMR parameters over time. However, we were able to relate longitudinal trajectories of multiple risk factors—in isolation and in combination—over 14 years to various CMR traits. Second, CMR measurements were obtained only in a subsample of the KORA cohort and the representativeness of this study sample for the initial cohort sample is limited. However, an analysis of cardiac parameters using statistical sampling weights to attain representativeness of the CMR sample to the full underlying cohort revealed no major differences to an unweighted analysis [43]. Third, we only identified three clusters for each risk factor and focussed on the most prevalent risk factors in our study sample. Fourth, except for the main analyses the results were not adjusted for multiple comparisons, thus weak associations remain questionable. And fifth, our sample was of European ancestry with unknown generalizability to other ethnic samples.

### Perspectives

We observed strong associations between risk factors levels measured over a 14-years’ time horizon with subclinical cardiac remodeling traits as assessed by CMR. These associations suggest that prevention of adverse cardiac remodelling should start early in life, since long term exposure to higher (multiple) risk factor levels demonstrated consistent and graded associations with adverse subclinical alterations of cardiac function and structure. Prevention strategies should include optimal control of modifiable risk factors, including BP, waist circumference, and LDL-C. Further studies should evaluate whether longitudinal risk factor information can be used to improve CVD risk prediction models for adverse cardiovascular remodelling and clinically overt CVD events.

## Conclusion

Long-term (14 years) exposure to elevated levels of to traditional CVD risk factors—modelled individually and conjointly—were associated with adverse CMR traits of cardiac structure and function. Longitudinal multivariable trajectory clusters added significant information to a multivariable risk profile obtained concurrent to the CMR examination (Table 4; Model B). Therefore, longitudinal multivariable trajectory clustering may allow better identification of individuals at high risk for cardiac remodelling. This premise deserves further investigation.

## Supplementary Information

**Additional file 1: Table S1.** Associations of individual cardiovascular risk factors from Exam 1–3 with MR traits of cardiac function and structure (Exam 3). **Table S2.** Associations of trajectory clusters for smoking pack-years and alcohol consumption (representing low, medium and high cumulative exposure to this risk factor over 14 years) with MR derived measures of cardiac structure and function. **Table S3.** Cross-sectional association of multivariable risk factor clusters from Exam 3 (representing low, medium and high exposure to 5 risk factors at this exam) with MR derived measures of cardiac structure and function (Model A); and a combined model displaying the association of multivariable risk factor clusters of Exam 3 (contemporaneous with the MRI) AND of the longitudinal (over 14-years) multivariable risk factor clusters with MR derived measures of cardiac structure and function (Model B). **Table S4.** Cross-sectional association of multivariable risk factor clusters from Exam 3 (representing low, medium and high exposure to 5 risk factors at this exam) with MR derived measures of cardiac structure and function (Model A); and a combined model displaying the association of multivariable risk factor clusters of Exam 3 (contemporaneous with the MRI) AND of the longitudinal (over 14-years) multivariable risk factor clusters with MR derived measures of cardiac structure and function (Model B). **Table S5.** Cross-sectional association of multivariable risk factor clusters from Exam 3 (representing low, medium and high exposure to 5 risk factors at this exam) with MR derived measures of cardiac structure and function (Model A); and a combined model displaying the association of multivariable risk factor clusters of Exam 3 (contemporaneous with the MRI) AND of the longitudinal (over 14-years) multivariable risk factor clusters with MR derived measures of cardiac structure and function (Model B).

## Data Availability

Access restrictions apply to the data underlying the findings and thus they cannot be made freely available. The data are subject to national data protection laws, and restrictions were imposed by the Ethics Committee of the Bavarian Chamber of Physicians, Munich, to ensure data privacy of the study participants as the informed consent given by KORA study participants does not cover data posting in public databases. However, data are available upon request by means of a project agreement from KORA (http://epi.helmholtz-muenchen.de/koragen/). Requests should be sent to kora.passt@helmholtz-muenchen.de and are subject to approval by the KORA Board.

## Abbreviations

BMI: Body mass index
BP: Blood pressure
BSA: Body surface area
bSSFP: Balanced steady state free precession
CMR: Cardiovascular magnetic resonance
CVD: Cardiovascular disease
HbA1c: Hemoglobin A1c
HDL-C: High density lipoprotein cholesterol
LDL-C: Low density lipoprotein cholesterol
LR: Likelihood ratio
LV: Left ventricle/left ventricular
RV: Right ventricle/right ventricular
WHR: Waist-hip-ratio
WHtR: Waist-height-ratio
