# Supplementary material for: Association of antecedent cardiovascular risk factor levels and trajectories with cardiovascular magnetic resonance-derived cardiac function and structure
Source: J Cardiovasc Magn Reson. 2021 Jan 4;23:2. doi: 10.1186/s12968-020-00698-w (PMC7780638; doi:10.1186/s12968-020-00698-w)
Supplement: Supplementary file 1 — Additional file 1: Table S1. Associations of individual cardiovascular risk factors from Exam 1–3 with MR traits of cardiac function and structure (Exam 3). Table S2. Associations of trajectory clusters for smoking pack-years and alcohol consumption (representing low, medium and high cumulative exposure to this risk factor over 14 years) with MR derived measures of cardiac structure and function. Table S3. Cross-sectional association of multivariable risk factor clusters from Exam 3 (representing low, medium and high exposure to 5 risk factors at this exam) with MR derived measures of cardiac structure and function (Model A); and a combined model displaying the association of multivariable risk factor clusters of Exam 3 (contemporaneous with the MRI) AND of the longitudinal (over 14-years) multivariable risk factor clusters with MR derived measures of cardiac structure and function (Model B). Table S4. Cross-sectional association of multivariable risk factor clusters from Exam 3 (representing low, medium and high exposure to 5 risk factors at this exam) with MR derived measures of cardiac structure and function (Model A); and a combined model displaying the association of multivariable risk factor clusters of Exam 3 (contemporaneous with the MRI) AND of the longitudinal (over 14-years) multivariable risk factor clusters with MR derived measures of cardiac structure and function (Model B). Table S5. Cross-sectional association of multivariable risk factor clusters from Exam 3 (representing low, medium and high exposure to 5 risk factors at this exam) with MR derived measures of cardiac structure and function (Model A); and a combined model displaying the association of multivariable risk factor clusters of Exam 3 (contemporaneous with the MRI) AND of the longitudinal (over 14-years) multivariable risk factor clusters with MR derived measures of cardiac structure and function (Model B). [file 12968_2020_698_MOESM1_ESM.docx]

**SUPPLEMENTAL MATERIAL**

**Supplemental Table 1** Associations of individual cardiovascular risk factors from Exam 1-3 with MR traits of cardiac function and structure (Exam 3)

|  | Left ventricle | | | | Right ventricle | | Cardiac fat | |
| --- | --- | --- | --- | --- | --- | --- | --- | --- |
| CV Risk factors | End-diastolic volume^†^ | Stroke volume^†^ | Myocardial mass, diastolic^†^ | Early diastolic filling rate | End-diastolic volume^†^ | Stroke volume^†^ | Epicardial fat, diastolic | Pericardial fat, diastolic |
| *KORA-Study* | β (95% CI) | β (95% CI) | β (95% CI) | β (95% CI) | β (95% CI) | β (95% CI) | β (95% CI) | β (95% CI) |
| **Exam 1** |  |  |  |  |  |  |  |  |
| Systolic BP | 0.10  (-0.08; 0.28) | **0.12***  **(0.00; 0.23)** | **0.23****  **(0.08; 0.39)** | 0.30  (-1.07; 1.66) | 0.14  (-0.07; 0.34) | 0.10  (-0.01; 0.21) | -0.02  (-0.07; 0.03) | 0.02  (-0.14; 0.18) |
| Diastolic BP | -0.20  (-0.47; 0.07) | **-0.18***  **(-0.35; -0.01)** | -0.10  (-0.33; 0.13) | -1.38  (-3.43; 0.68) | -0.28  (-0.59; 0.02) | **-0.18***  **(-0.35; -0.01)** | **0.10****  **(0.03; 0.17)** | 0.16  (-0.08; 0.4) |
| WC | -0.16  (-0.33; 0.01) | **-0.12***  **(-0.23; -0.01)** | 0.14  (0.00; 0.29) | -0.19  (-1.48; 1.1) | **-0.23***  **(-0.43; -0.04)** | **-0.12***  **(-0.23; -0.01)** | **0.11*****  **(0.06; 0.15)** | **0.47*****  **(0.31; 0.62)** |
| HbA1c | -2.12  (-6.24; 2.01) | -1.90  (-4.51; 0.71) | 0.56  (-2.92; 4.04) | -12.91  (-43.97; 18.14) | -3.44  (-8.01; 1.14) | -1.41  (-3.96; 1.13) | -0.22  (-1.29; 0.84) | 1.24  (-2.43; 4.91) |
| LDL-C | **-0.06****  **(-0.11; -0.02)** | **-0.05****  **(-0.07; -0.02)** | -0.01  (-0.05; 0.02) | **-0.35***  **(-0.66; -0.03)** | **-0.08****  **(-0.12; -0.03)** | **-0.04****  **(-0.06; -0.01)** | 0.00  (-0.01; 0.01) | 0.01  (-0.03; 0.05) |
|  | R^2^=0.11 | R^2^=0.13 | R^2^=0.25 | R^2^=0.12 | R^2^=0.19 | R^2^=0.12 | R^2^=0.29 | R^2^=0.39 |
| **Exam 2** |  |  |  |  |  |  |  |  |
| Systolic BP | 0.15  (-0.02; 0.33) | **0.16****  **(0.05; 0.27)** | **0.33*****  **(0.18; 0.48)** | 0.22  (-1.10; 1.54) | 0.17  (-0.03; 0.37) | **0.12***  **(0.01; 0.23)** | **-0.05***  **(-0.10; -0.01)** | -0.08  (-0.23; 0.08) |
| Diastolic BP | **-0.36***  **(-0.65; -0.07)** | **-0.28****  **(-0.46; -0.10)** | **-0.36****  **(-0.61; -0.11)** | -1.97  (-4.18; 0.23) | **-0.45****  **(-0.78; -0.12)** | **-0.23***  **(-0.41; -0.05)** | **0.11****  **(0.03; 0.18)** | 0.25  (-0.01; 0.51) |
| WC | **-0.21****  **(-0.35; -0.07)** | -**0.16****  **(-0.25; -0.07)** | 0.07  (-0.05; 0.19) | -0.91  (-1.98; 0.16) | **-0.29*****  **(-0.45; -0.13)** | **-0.16****  **(-0.24; -0.07)** | **0.12*****  **(0.08; 0.15)** | **0.50*****  **(0.38; 0.62)** |
| HbA1c | -1.99  (-5.68; 1.69) | -2.09  (-4.40; 0.22) | -0.06  (-3.20; 3.09) | -4.49  (-32.44; 23.47) | **-4.31***  **(-8.60; -0.01)** | -1.32  (-3.69; 1.05) | 0.39  (-0.54; 1.32) | 1.02  (-2.1; 4.15) |
| LDL-C | **-0.06***  **(-0.11; -0.01)** | **-0.04****  **(-0.07; -0.01)** | 0.01  (-0.03; 0.05) | -0.28  (-0.63; 0.08) | **-0.05***  **(-0.11; 0.00)** | -0.03  (-0.06; 0.00) | 0.00  (-0.01; 0.01) | 0.00  (-0.04; 0.04) |
|  | R^2^=0.14 | R^2^=0.16 | R^2^=0.27 | R^2^=0.14 | R^2^=0.19 | R^2^=0.13 | R^2^=0.31 | R^2^=0.43 |
| **Exam 3** |  |  |  |  |  |  |  |  |
| Systolic BP | 0.04  (-0.13; 0.22) | **0.11***  **(0.01; 0.22)** | **0.30*****  **(0.15; 0.44)** | -0.23  (-1.49; 1.04) | 0.11  (-0.08; 0.31) | 0.10  (0.00; 0.21) | -0.01  (-0.05; 0.03) | 0.03  (-0.11; 0.17) |
| Diastolic BP | -0.19  (-0.45; 0.08) | **-0.24****  **(-0.40; -0.07)** | -0.20  (-0.42; 0.02) | -1.84  (-3.80; 0.13) | **-0.36***  **(-0.66; -0.06)** | **-0.23****  **(-0.40; -0.07)** | 0.03  (-0.04; 0.09) | -0.03  (-0.25; 0.20) |
| WC | **-0.28*****  **(-0.42; -0.15)** | **-0.20*****  **(-0.28; -0.11)** | 0.01  (-0.10; 0.12) | **-1.11***  **(-2.10; -0.13)** | **-0.37*****  **(-0.52; -0.22)** | **-0.18*****  **(-0.26; -0.10)** | **0.13*****  **(0.10; 0.17)** | **0.55*****  **(0.43; 0.66)** |
| HbA1c | -0.18  (-2.92; 2.55) | **-1.71***  **(-3.40; -0.02)** | **3.54****  **(1.29; 5.79)** | -12.50  (-32.57; 7.57) | -0.88  (-3.90; 2.14) | -1.20  (-2.87; 0.46) | -0.27  (-0.94; 0.41) | 1.05  (-1.17; 3.26) |
| LDL-C | **-0.08****  **(-0.13; -0.03)** | **-0.05****  **(-0.08; -0.02)** | -0.02  (-0.06; 0.02) | -0.37  (-0.74; 0.00) | **-0.08****  **(-0.14; -0.03)** | **-0.04***  **(-0.07; -0.01)** | 0.00  (-0.01; 0.01) | 0.01  (-0.03; 0.06) |
|  | R^2^=0.13 | R^2^=0.19 | R^2^=0.29 | R^2^=0.17 | R^2^=0.22 | R^2^=0.16 | R^2^=0.33 | R^2^=0.47 |

Data are β-coefficients (with 95% confidence interval; indicating change in outcome variable per 1 unit increment of risk factor) from multivariable linear regression models adjusted for age, sex, smoking status, alcohol consumption, physical activity, antihypertensive medication,

lipid-lowering medication, glucose-lowering medication; ^***^p<0.001; ^**^p<0.01; ^*^p<0.05, ^†^indexed to body surface area;

**Supplemental Table 2** Associations of trajectory clusters for **smoking pack-years and alcohol consumption** (representing low, medium and high cumulative exposure to this risk factor over 14 years) with MR derived measures of cardiac structure and function.

|  |  | Left ventricle | | | | Right ventricle | | Cardiac fat | |
| --- | --- | --- | --- | --- | --- | --- | --- | --- | --- |
| CV Risk factor  Clusters^†^ | N | End-diastolic volume^‡^ | Stroke volume^‡^ | Myocardial mass, diastolic^‡^ | Early diastolic filling rate | End-diastolic volume^‡^ | Stroke volume^‡^ | Epicardial fat, diastolic | Pericardial fat, diastolic |
|  | 349 | β (95% CI) | β (95% CI) | β (95% CI) | β (95% CI) | β (95% CI) | β (95% CI) | β (95% CI) | β (95% CI) |
| Pack-years (low) | 240 | Ref. | Ref. | Ref. | Ref. | Ref. | Ref. | Ref. | Ref. |
| (middle) | 92 | -0.65  (-4.86; 3.56) | -1.40  (-4.00; 1.21) | **3.76***  **(0.28; 7.23)** | -22.53  (-53.45; 8.38) | -2.62  (-7.28; 2.05) | -1.14  (-3.69; 1.41) | 0.59  (-0.48; 1.65) | 0.78  (-2.70; 4.26) |
| (high) | 17 | -3.88  (-11.62; 3.86) | -2.34  (-7.13; 2.45) | -1.66  (-8.05; 4.73) | -12.19  (-69.05; 44.66) | -6.79  (-15.86; 2.29) | -1.98  (-6.95; 2.98) | 1.21  (-0.68; 3.10) | **6.78***  **(0.58; 12.98)** |
| Alcohol cons. (low) | 232 | Ref. | Ref. | Ref. | Ref. | Ref. | Ref. | Ref. | Ref. |
| (middle) | 93 | 2.04  (-2.85; 6.93) | 0.54  (-2.49; 3.56) | 2.23  (-1.74; 6.20) | 12.09  (-23.62; 47.80) | 3.06  (-2.61; 8.73) | 1.21  (-1.87; 4.30) | 0.17  (-1.04; 1.38) | 1.42  (-2.56; 5.4) |
| (high) | 24 | 6.03  (-3.32; 15.38) | 2.22  (-3.57; 8.01) | 5.14  (-2.45; 12.73) | 26.11  (-42.17; 94.40) | 8.08  (-2.49; 18.65) | 2.95  (-2.80; 8.70) | -0.62  (-2.90; 1.65) | 3.37  (-4.1; 10.85) |

Data are β-coefficients (with 95% confidence interval; indicating change in outcome variable between reference cluster and risk factor cluster) from multivariable linear regression models adjusted for the other risk factor respectively and for age, sex, systolic and diastolic BP, WC, HbA1c, LDL-C, physical activity, antihypertensive medication, lipid-lowering medication, glucose-lowering medication measured in Exam 3; ^***^p<0.001; ^**^p<0.01; ^*^p<0.05, ^†^clusters were calculated by k-means clustering, ^‡^indexed to body surface area; Ref. = reference cluster

**Supplemental Table 3** Cross-sectional association of **multivariable risk factor clusters from Exam 3** (representing low, medium and high exposure to 5 risk factors at this exam) with MR derived measures of cardiac structure and function (**Model A**); and a combined model displaying the association of **multivariable risk factor clusters of Exam 3** (contemporaneous with the MRI) AND of the **longitudinal (over 14-years) multivariable risk factor clusters** with MR derived measures of cardiac structure and function (**Model B**).

|  |  | Left ventricle | | | | Right ventricle | | Cardiac fat | |
| --- | --- | --- | --- | --- | --- | --- | --- | --- | --- |
| CV Risk factors | N | End-diastolic volume^†^ | Stroke volume^†^ | Myocardial mass, diastolic^†^ | Early diastolic filling rate | End-diastolic volume^†^ | Stroke volume^†^ | Epicardial fat, diastolic | Pericardial fat, diastolic |
|  | 349 | β (95% CI) | β (95% CI) | β (95% CI) | β (95% CI) | β (95% CI) | β (95% CI) | β (95% CI) | β (95% CI) |
| *Model A* |  |  |  |  |  |  |  |  |  |
| Cross-sectional |  |  |  |  |  |  |  |  |  |
| multivariable risk factor clusters^§^ |  |  |  |  |  |  |  |  |  |
| (low) | 167 | Ref. | Ref. | Ref. | Ref. | Ref. | Ref. | Ref. | Ref. |
| (middle) | 131 | **-5.95****  **(-9.46; -2.44)** | **-4.57*****  **(-6.78; -2.35)** | 0.95  (-1.97; 3.86) | **-41.87****  **(-67.4; -16.35)** | **-5.96****  **(-9.99; -1.93)** | **-3.14****  **(-5.33; -0.96)** | 0.78  (-0.17; 1.73) | **4.51****  **(1.27; 7.75)** |
| (high) | 51 | **-5.49***  **(-10.39; -0.59)** | **-3.90***  **(-7.00; -0.80)** | 1.04  (-3.03; 5.12) | **-41.26***  **(-76.95; -5.57)** | **-6.94***  **(-12.61; -1.26)** | **-3.48***  **(-6.55; -0.41)** | 0.79  (-0.51; 2.09) | **4.69***  **(0.27; 9.12)** |
| *Model B* |  |  |  |  |  |  |  |  |  |
| Cross-sectional |  |  |  |  |  |  |  |  |  |
| multivariable risk factor clusters^§^ |  |  |  |  |  |  |  |  |  |
| (low) | 167 | Ref. | Ref. | Ref. | Ref. | Ref. | Ref. | Ref. | Ref. |
| (middle) | 131 | -3.68  (-7.48; 0.11) | **-3.58****  **(-5.99; -1.17)** | 1.62  (-1.57; 4.82) | -26.10  (-53.61; 1.41) | -4.26  (-8.57; 0.06) | -2.28  (-4.65; 0.09) | 0.64  (-0.37; 1.65) | **4.02***  **(0.65; 7.38)** |
| (high) | 51 | -3.34  (-8.32; 1.64) | -2.87  (-6.03; 0.29) | 1.31  (-2.88; 5.51) | -25.63  (-61.72; 10.46) | -5.03  (-10.78; 0.71) | -2.57  (-5.73; 0.59) | 0.49  (-0.79; 1.78) | 3.53  (-0.75; 7.81) |
| Longitudinal |  |  |  |  |  |  |  |  |  |
| multivariable risk factor trajectory clusters^‡^ |  |  |  |  |  |  |  |  |  |
| (low) | 133 | Ref. | Ref. | Ref. | Ref. | Ref. | Ref. | Ref. | Ref. |
| (middle) | 139 | **-7.90*****  **(-12.15; -3.65)** | **-4.07****  **(-6.76; -1.37)** | 0.10  (-3.48; 3.68) | **-59.64*****  **(-90.43; -28.84)** | **-8.28****  **(-13.15; -3.41)** | **-3.61****  **(-6.29; -0.93)** | **1.63****  **(0.50; 2.76)** | **6.55****  **(2.80; 10.3)** |
| (high) | 77 | **-6.96****  **(-11.48; -2.44)** | **-4.67****  **(-7.53; -1.8)** | **4.30***  **(0.49; 8.11)** | **-60.57*****  **(-93.35; -27.8)** | **-11.43*****  **(-16.55; -6.30)** | **-4.20****  **(-7.02; -1.38)** | **3.29*****  **(2.10; 4.47)** | **13.7*****  **(9.77; 17.63)** |
|  |  |  |  |  |  |  |  |  |  |
| LR-test^§^ |  | p<0.001 | p=0.002 | p=0.036 | p<0.001 | p<0.001 | p=0.005 | p<0.001 | p<0.001 |

^§^Multivariable clusters included the risk factors systolic BP, diastolic BP, WC, HbA1c, LDL-C, pack-years and alcohol consumption and were calculated by k-means clustering.

Data are β-coefficients (with 95% confidence interval; indicating change in outcome variable between reference cluster and risk factor cluster) from multivariable linear regression models adjusted for age, sex, physical activity, antihypertensive medication, lipid-lowering medication, glucose-lowering medication measured in Exam 3; ^***^p<0.001; ^**^p<0.01; ^*^p<0.05, ^†^indexed to body surface area; Ref. = reference cluster;; ^‡^Likelihood-Ratio-test for adding the longitudinal multivariable risk factor trajectory clusters to the cross-sectional multivariable risk factor clusters (*Model B*).

**Supplemental Table 4** Cross-sectional association of **multivariable risk factor clusters from Exam 3** (representing low, medium and high exposure to 5 risk factors at this exam) with MR derived measures of cardiac structure and function (**Model A**); and a combined model displaying the association of **multivariable risk factor clusters of Exam 3** (contemporaneous with the MRI) AND of the **longitudinal (over 14-years) multivariable risk factor clusters** with MR derived measures of cardiac structure and function (**Model B**).

|  |  | Left ventricle | | | | Right ventricle | |
| --- | --- | --- | --- | --- | --- | --- | --- |
| CV Risk factors | N | End-systolic volume^†^ | Ejection Fraction | Myocardial mass, systolic^†^ | Peak ejection rate | End-systolic volume^†^ | Ejection Fraction |
|  | 349 | β (95% CI) | β (95% CI) | β (95% CI) | β (95% CI) | β (95% CI) | β (95% CI) |
| *Model A* |  |  |  |  |  |  |  |
| Cross-sectional |  |  |  |  |  |  |  |
| multivariable risk factor clusters^§^ |  |  |  |  |  |  |  |
| (low) | 104 | Ref. | Ref. | Ref. | Ref. | Ref. | Ref. |
| (middle) | 164 | -0.56  (-2.78; 1.66) | 0.01  (-1.99; 2.00) | 1.08  (-2.27; 4.43) | -9.01  (-42.26; 24.24) | -0.65  (-3.47; 2.17) | -0.44  (-2.14; 1.27) |
| (high) | 81 | -1.52  (-4.19; 1.14) | -0.51  (-2.90; 1.88) | 2.88  (-1.13; 6.9) | -35.33  (-75.22; 4.57) | **-3.86***  **(-7.27; -0.46)** | -0.02  (-2.08; 2.04) |
| *Model B* |  |  |  |  |  |  |  |
| Cross-sectional |  |  |  |  |  |  |  |
| multivariable risk factor clusters^§^ |  |  |  |  |  |  |  |
| (low) | 104 | Ref. | Ref. | Ref. | Ref. | Ref. | Ref. |
| (middle) | 164 | 0.25  (-2.16; 2.66) | 0.02  (-2.15; 2.19) | -0.26  (-3.86; 3.35) | 2.74  (-33.14; 38.61) | 0.66  (-2.38; 3.69) | -0.59  (-2.44; 1.27) |
| (high) | 81 | -0.46  (-3.39; 2.48) | -0.48  (-3.13; 2.16) | 1.10  (-3.29; 5.48) | -19.62  (-63.27; 24.04) | -2.14  (-5.85; 1.57) | -0.22  (-2.48; 2.05) |
| Longitudinal |  |  |  |  |  |  |  |
| multivariable risk factor trajectory clusters^‡^ |  |  |  |  |  |  |  |
| (low) | 83 | Ref. | Ref. | Ref. | Ref. | Ref. | Ref. |
| (middle) | 159 | -2.16  (-4.94; 0.63) | 0.08  (-2.43; 2.59) | 2.41  (-1.75; 6.57) | -20.36  (-61.79; 21.07) | -3.19  (-6.71; 0.33) | 0.46  (-1.69; 2.61) |
| (high) | 107 | -2.91  (-6.39; 0.56) | -0.31  (-3.44; 2.82) | **7.05****  **(1.85; 12.25)** | **-63.52***  **(-115.28; -11.76)** | **-5.48***  **(-9.85; -1.10)** | 0.44  (-2.24; 3.11) |
|  |  |  |  |  |  |  |  |
| LR-test^§^ |  | p=0.215 | p=0.938 | p=0.014 | p=0.027 | p=0.043 | p=0.912 |

^§^Multivariable clusters included the risk factors systolic BP, diastolic BP, WC, HbA1c and LDL-C and were calculated by k-means clustering.

Data are β-coefficients (with 95% confidence interval; indicating change in outcome variable between reference cluster and risk factor cluster) from multivariable linear regression models adjusted for age, sex, smoking status, alcohol consumption, physical activity, antihypertensive medication, lipid-lowering medication, glucose-lowering medication measured in Exam 3; ^***^p<0.001; ^**^p<0.01; ^*^p<0.05, ^†^indexed to body surface area; Ref. = reference cluster;; ^‡^Likelihood-Ratio-test for adding the longitudinal multivariable risk factor trajectory clusters to the cross-sectional multivariable risk factor clusters (*Model B*).

**Supplemental Table 5** Cross-sectional association of **multivariable risk factor clusters from Exam 3** (representing low, medium and high exposure to 5 risk factors at this exam) with MR derived measures of cardiac structure and function (**Model A**); and a combined model displaying the association of **multivariable risk factor clusters of Exam 3** (contemporaneous with the MRI) AND of the **longitudinal (over 14-years) multivariable risk factor clusters** with MR derived measures of cardiac structure and function (**Model B**).

|  |  | Left ventricle | | | | Right ventricle | | Cardiac fat | |
| --- | --- | --- | --- | --- | --- | --- | --- | --- | --- |
| CV Risk factors | N | End-diastolic volume^†^ | Stroke volume^†^ | Myocardial mass, diastolic^†^ | Early diastolic filling rate | End-diastolic volume^†^ | Stroke volume^†^ | Epicardial fat, diastolic | Pericardial fat, diastolic |
|  | 349 | β (95% CI) | β (95% CI) | β (95% CI) | β (95% CI) | β (95% CI) | β (95% CI) | β (95% CI) | β (95% CI) |
| *Model A* |  |  |  |  |  |  |  |  |  |
| Cross-sectional |  |  |  |  |  |  |  |  |  |
| multivariable risk factor clusters^§^ |  |  |  |  |  |  |  |  |  |
| (low) | 104 | Ref. | Ref. | Ref. | Ref. | Ref. | Ref. | Ref. | Ref. |
| (middle) | 164 | -2.88  (-6.63; 0.86) | -2.14  (-4.53; 0.25) | -0.59  (-3.70; 2.52) | -22.54  (-49.89; 4.81) | -2.02  (-6.24; 2.20) | -1.55  (-3.86; 0.77) | -0.46  (-1.45; 0.54) | 0.79  (-2.68; 4.26) |
| (high) | 81 | **-6.03****  **(-10.44; -1.62)** | **-4.47****  **(-7.29; -1.66)** | 1.27  (-2.40; 4.93) | **-45.87****  **(-78.07; -13.68)** | **-6.69****  **(-11.70; -1.68)** | **-3.56***  **(-6.30; -0.81)** | -0.05  (-1.21; 1.11) | 2.16  (-1.89; 6.22) |
| *Model B* |  |  |  |  |  |  |  |  |  |
| Cross-sectional |  |  |  |  |  |  |  |  |  |
| multivariable risk factor clusters^§^ |  |  |  |  |  |  |  |  |  |
| (low) | 104 | Ref. | Ref. | Ref. | Ref. | Ref. | Ref. | Ref. | Ref. |
| (middle) | 164 | -0.71  (-4.65; 3.24) | -0.62  (-3.13; 1.88) | -1.41  (-4.70; 1.89) | -9.87  (-38.68; 18.94) | 0.10  (-4.32; 4.51) | -0.39  (-2.82; 2.03) | **-1.27***  **(-2.32; -0.22)** | -1.56  (-5.21; 2.09) |
| (high) | 81 | -3.52  (-8.18; 1.14) | -2.73  (-5.69; 0.23) | 0.44  (-3.45; 4.33) | -31.86  (-65.88; 2.15) | -4.35  (-9.59; 0.89) | -2.27  (-5.15; 0.61) | -0.96  (-2.18; 0.25) | -0.41  (-4.66; 3.85) |
| Longitudinal |  |  |  |  |  |  |  |  |  |
| multivariable risk factor trajectory clusters^‡^ |  |  |  |  |  |  |  |  |  |
| (low) | 83 | Ref. | Ref. | Ref. | Ref. | Ref. | Ref. | Ref. | Ref. |
| (middle) | 159 | **-6.15****  **(-10.7; -1.6)** | **-4.30****  **(-7.19; -1.41)** | 1.48  (-2.32; 5.28) | -31.49  (-64.7; 1.73) | **-5.97***  **(-11.09; -0.86)** | **-3.32***  **(-6.13; -0.51)** | **2.24*****  **(1.03; 3.46)** | **5.87****  **(1.64; 10.1)** |
| (high) | 107 | **-9.47****  **(-14.92; -4.03)** | **-6.54*****  **(-10.0; -3.08)** | **5.78***  **(1.23; 10.32)** | **-66.99****  **(-106.74; -27.24)** | **-10.81****  **(-16.93; -4.69)** | **-5.69****  **(-9.06; -2.33)** | **3.09*****  **(1.66; 4.52)** | **10.91*****  **(5.92; 15.89)** |
|  |  |  |  |  |  |  |  |  |  |
| LR-test^§^ |  | p=0.002 | p<0.001 | p=0.015 | p=0.003 | p=0.002 | p=0.003 | p<0.001 | p<0.001 |

^§^Multivariable clusters included the risk factors systolic BP, diastolic BP, WC, HbA1c and LDL-C and were calculated by k-means clustering.

Data are β-coefficients (with 95% confidence interval; indicating change in outcome variable between reference cluster and risk factor cluster) from multivariable linear regression models adjusted for age, sex, smoking status, alcohol consumption, physical activity, antihypertensive medication, lipid-lowering medication, glucose-lowering medication measured in Exam 1; ^***^p<0.001; ^**^p<0.01; ^*^p<0.05, ^†^indexed to body surface area; Ref. = reference cluster;; ^‡^Likelihood-Ratio-test for adding the longitudinal multivariable risk factor trajectory clusters to the cross-sectional multivariable risk factor clusters (*Model B*).
